# Annihilation of the Somali upwelling system during summer monsoon

**DOI:** 10.1038/s41598-019-44099-1

**Published:** 2019-05-20

**Authors:** Abhisek Chatterjee, B. Praveen Kumar, Satya Prakash, Prerna Singh

**Affiliations:** ESSO- Indian National Centre for Ocean Information Services, Hyderabad, India

**Keywords:** Projection and prediction, Physical oceanography

## Abstract

Somali upwelling system during northern summer is believed to be the largest upwelling region in the Indian Ocean and has motivated some of the early studies on the Indian Ocean. Here we present results from observations and ocean model to show that the upwelling along the Somali coast is limited to the early phase of the summer monsoon and later primarily limited to the eddy dominated flows in the northern and some extent in the southern part of the coast. Major part of the Somali coast (~60% of the entire coastal length) shows prominent downwelling features driven by offshore negative windstress curl and subsurface entrainment mixing. Further, we show that the surface cooling of coastal waters are dominantly driven by subsurface entrainment and surface heat fluxes. These findings not only augment the existing knowledge of the Somali upwelling system, but also have serious implications on the regional climate. Most importantly, our analysis underscores the use of alongshore winds only to project future (climate driven) changes in the upwelling intensity along this coast.

## Introduction

**Figure 1:**
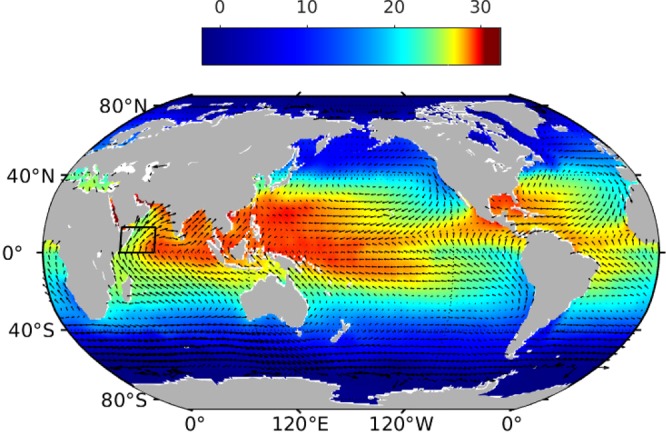
Map of global climatological SST (shading) from NIOA^26^ and surface winds (vector) from QuikSCAT for the month of July. The four eastern boundary upwelling systems in the Pacific and Atlantic are well marked by the cold surface temperature. The only a similar, albeit weaker and smaller, upwelling is seen along the coast of Somalia and Arabia in the western Arabian Sea. The black box is representing the coast of Somalia.

Somali upwelling system is considered to be the fifth largest upwelling system of the global ocean and the strongest in the Indian Ocean [Fig. 1]. In contrast to the other eastern boundary upwelling systems, Somali current is an upwelling system associated with western boundary current and reverses its direction annually owing to the seasonally reversing monsoon winds^1–3^. During summer monsoon Somali current flows poleward and exhibits a maximum transport of ~37 ± 5Sv^4^. This is also marked by a strong surface cooling along the coast, exhibiting strong zonal SST gradient across the Arabian Sea which helps to enhance the moisture transport into the Asian subcontinent and therefore, plays a role in the summer monsoon rainfall over this region^5^. Summer upwelling also plays an important role in this region’s ecology by elevating nutrient concentration in the upper surface layer, thus making it as one of the most productive marine ecosystems in the world oceans^6–12^.

**Figure 2:**
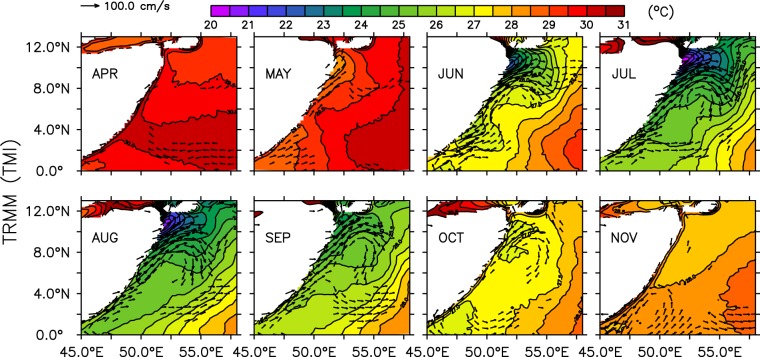
Monthly climatological sea surface temperature from TRMM (shaded) and surface currents from OSCAR (vectors) showing evolution SST along the Somali coast. Currents of magnitude less than 50 cm s^−1^ are masked out and plotted for every alternate grids. Note also that the climatologies are created based on data from the year 1998 to 2008 owing to the availability of TRMM TMI data.

Since the first International Indian Ocean expedition (IIOE) in 1960′s, dynamics of this current system is being studied extensively by many researchers. It is now well known that Somali coast experiences a low level southwesterly surface jet during summer, called as Findlater Jet^13^, that drives the surface currents northward^1,2,14,15^. Climatological data (Figs 2 and S1) indicate that east African currents, likely forced by the easterly winds in the southern hemisphere^16^, cross the equator in early May and flow northward along the Somali coast. As the alongshore winds start to peak in May, the current extend further to ~3–4°N and then separates from the coast and flow offshore to form an anti-cyclonic gyre, frequently referred as the Southern Gyre^17^. Subsequently a cold wedge forms along the northern offshore flank of the Gyre^7^. At the same time, coastal currents continue to extend northward and another anti-cyclonic gyre, known as Great Whirl, forms with a cold wedge north of 8°N along its northern offshore flow^3^ (henceforth, the Southern Gyre and Great Whirl are referred as SG and GW, respectively). As the monsoon progresses, currents in the GW strengthen further to reach up to 250 cm/s during July^18^ and is by then comparable with the other strongest western boundary currents such as Gulf Stream and Kuroshio. The SG disappears or weakens considerably by August, but, the GW still maintains its strength. Finally, as the summer monsoon winds start to withdraw from the north Indian Ocean during September, strength of the Somali current weakens considerably. However, GW survives for another month after the Findlater Jet wanes off along the Somali coast^18^.

Despite its uniqueness, the dynamics of the formation of these two gyre systems is still not well understood. Previous studies suggest that non-linearity associated with the strong cross-equatorial currents plays an important role in the formation of the SG^1^, whereas, strong Findlater Jet north of the equator drives the formation of GW^16^. At this time, surface temperature all along the Somali coast decreases rapidly by at least 5 °C and at some places it even decreases to ~20 °C, which is previously attributed primarily to the classical coastal upwelling owing to the offshore Ekman transport driven by the alongshore winds.

**Figure 3:**
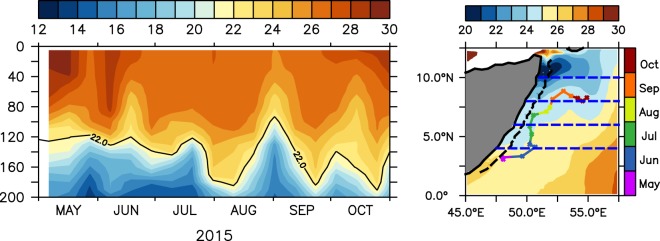
(**a**) Temperature time-series based on an ARGO float during summer of 2015. (**b**) Track of the ARGO float is marked by the colour curve with a scale (right) representing month. Monthly climatological SST for July from TRMM TMI is shaded with a scale (above). The dashed black line and blue lines represents the alongshore sections along 1000 m isobath and various latitudinal sections off the coast of Somalia.

**Figure 4:**
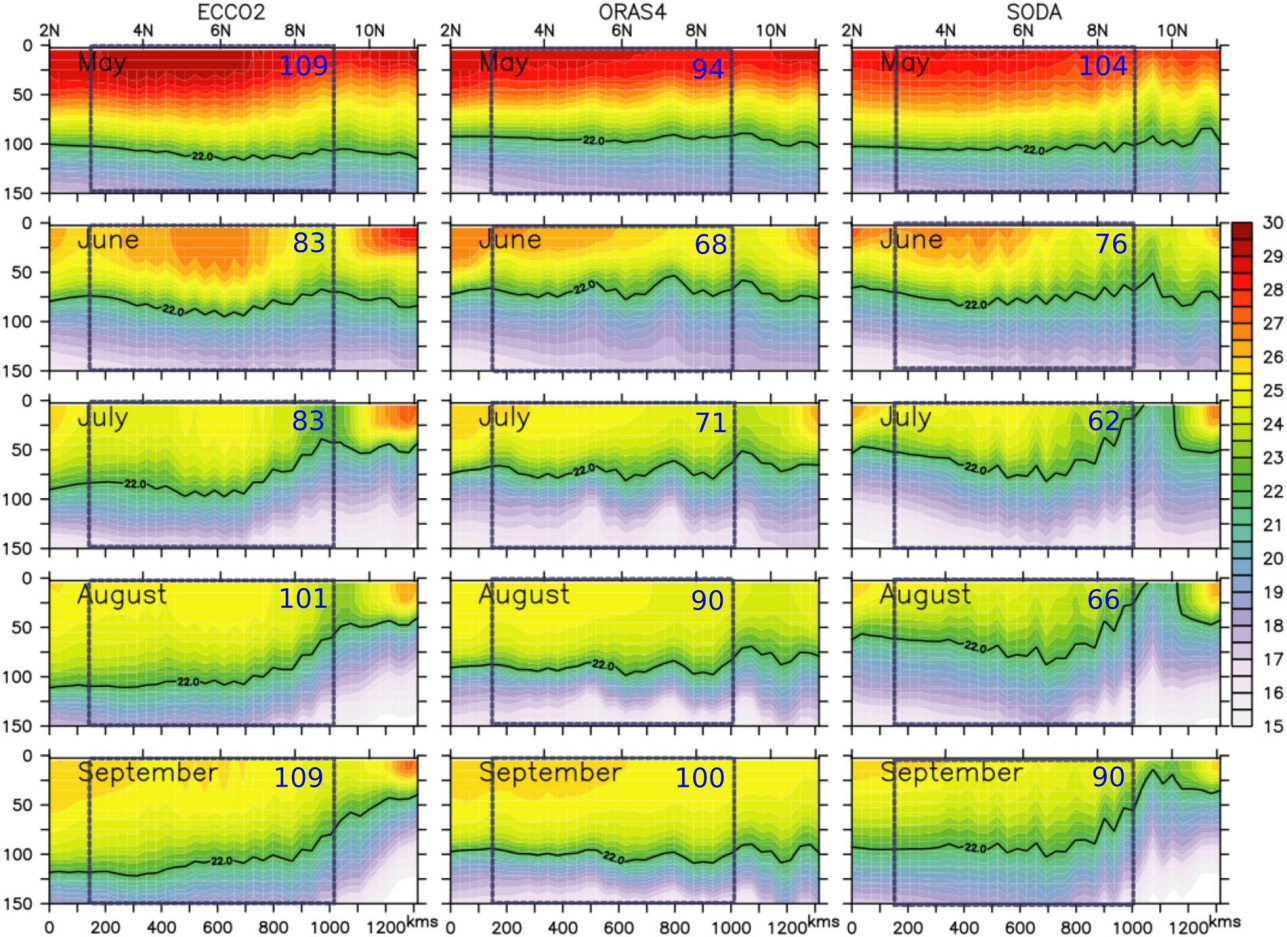
Evolution of thermocline off Somalia coast during summer from ECCO2, ORAS4 and SODA. The blue dotted box represents central part of the alongshore section where the deepening of the thermocline is evident. The numbers in blue fonts represent the depth of the D22 averaged over the box.

A majority of these analyses on coastal upwelling focused mostly on the alongshore wind driven offshore Ekman transport and the associated Sea Surface Temperature (SST) cooling signatures^19^. However, as the Somali coast is one of the data sparse regions owing to the piracy problem, direct *in-situ* observations to verify the upwelling features are extremely rare. Interestingly, an ARGO float (WMO ID 1901517) crossed the Somali coast during summer of 2015 and allowed an unprecedented opportunity to look at the temporal structure of the thermocline along the coast (Fig. 3). The float was at 3°N in May and traveled along the Somali coast and finally got advected offshore sometime in September. During its entire course the float was within the cold SST regime off Somalia, but, surprisingly, along its entire trajectory, upwelling was seen only during early September when it reached the cold wedge along the northern flank of the GW, otherwise the 22 °C isotherm (D22–a proxy for the thermocline depth) always remained deeper than 120 m. In order to confirm it further, we looked at the popular ocean reanalysis products (ECCO^20^, ORAS4^21^ and SODA^22,23^) along an alongshore section on the shelf-break (1000 m bathemetry) off Somalia (dashed black contour in Fig. 3). A comparison of temperature along the alongshore section from all three reanalysis data suggest a similar feature as observed by the ARGO float (Fig. 4). The upwelling is prominent only to the north of 9°N and a weaker uplifting of thermocline can be seen at ~2–3°N in the early phase of the summer monsoon. Noticeably in the central part (marked by the dashed box in Fig. 4) D22 remains relatively much deeper in all three data sets. However, thermocline in this part does shallow up by about 25 m during the early phase of the summer monsoon (June and July), but as the monsoon peaks in August, it gets restored in the deeper depths. Interestingly, the upper water column cools progressively as the thermocline deepens over the course of the summer months. In other words, despite the prevailing upwelling favorable alongshore winds the upwelling along the coast off Somalia is primarily limited in the northern part of the section north of 9°N and also to a small section between 2–3°N; a major portion of the Somali coast (~60% of the entire coastal length) exhibits strong downwelling signatures. This contradicts the existing perception about the Somali upwelling system built over the last five decades.

In order to understand the observed deepening of thermocline and the evolution of cold surface temperature, in the absence of any other observations, in the next section, we have analysed simulations from an Ocean General Circulation Model (OGCM) (described in Methodology Section) and frequently discuss a vertical section approximately along the shelf break (~1000 m isobath) along the coastline (dashed black curve in Fig. 3). Notably, the results presented here are robust and independent of the section chosen along a slightly different isobath. Also, note here that the results are discussed based on climatology prepared using the interannual simulation of the model for 1993–2014 period.

## Results

**Figure 5:**
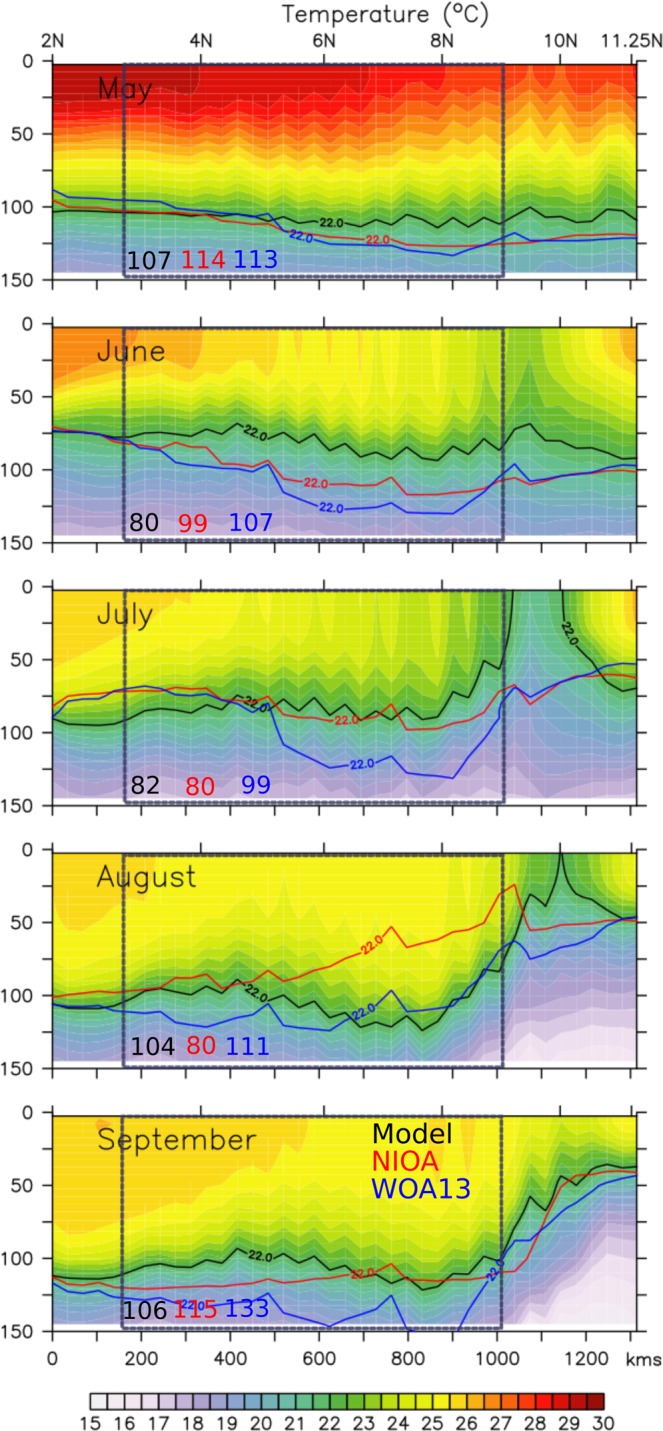
Alongshore section of model simulated temperature along the 1000 m isobath off Somalia. The black, red and blue contour represent D22 isotherm from model, NIOA and WOA13, respectively. As in Fig. 4, the blue dotted box represents C_SEC_ and the numbers in black, red and blue fonts represent the depth of the D22 averaged over the blue box from model, NIOA and WOA13, respectively.

Figure 5 compares the model simulated temperature section along the ~1000 m isobath off Somalia with two gridded observation datasets: the World Ocean Atlas 2013 (WOA13; Locarnini *et al*.^24^ and Zweng *et al*.^25^) and the North Indian Ocean Atlas (NIOA; Chatterjee *et al*.^26^). The simulated seasonal evolution of thermocline off Somalia compares well with the observations (Fig. 5) and that from the reanalysis products (Fig. 4). As seen in the observations, the model simulated thermocline also deepened significantly over the course of the monsoon season in the central stretch of the coast (~3–9°; henceforth referred as central section or C_SEC_), sandwiched between two upwelling zones: a weaker one in the south (south of 3°N or S_SEC_) and a much stronger in the north (north of 9°N or N_SEC_). Note, however, that intensity of upwelling in the northern part of the section is weaker in the observations compared to the model simulation possibly due to the lack of enough observed profiles in the gridded observation data in this region and rigid smoothing that are imposed while gridding these datasets. Nonetheless, thermocline lifts up north of 9°N and to a lesser extent between 2–3°N, suggesting signatures of upwelling and shoals down between the vast swath of these upwelling zones in both the observations and model simulations, though their magnitude mismatches. Interestingly, the strong upwelling in the northern part too (associated with GW) starts weakening from August when the D22 barely penetrates into the surface and by September D22 deepened to about 50 m depth in the northern part and more than 100 m in the rest of the section.

**Figure 6:**
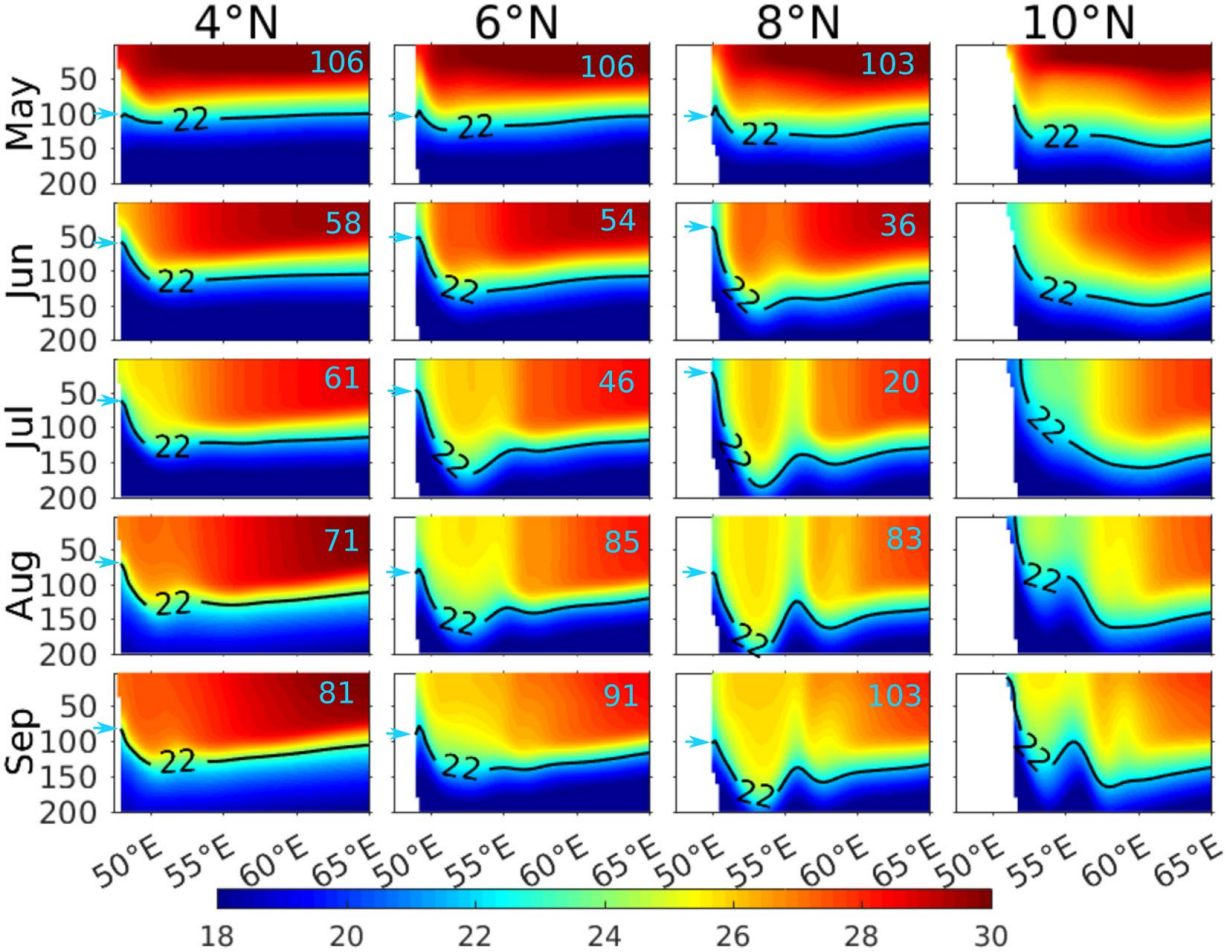
Vertical sections of temperature along 4°N, 6°N, 8°N and 10°N off the Somalia coast during May–September. The black contours represent D22 and the cyan fonts represent the depth of the D22 right adjacent to the coast marked by cyan arrow.

In order to better document the temperature evolution of the upper water column of this region, we also looked at the near crosshore sections of temperature (Fig. 6) during May to September along the 4°N, 6°N, 8°N and 10°N latitude belts off Somalia coast (dashed blue lines in Fig. 3). During May, when the alongshore winds are weakest, surface temperature reaches close to 30 °C and the thermocline along the Somali coast remains relatively flat at ~100 m depth except at very close to the coast where wind driven coastal upwelling is evident. This upwelling signatures strengthen from south to north with D22 shoals from 100 m at 4°N to 75 m at 10°N. This weaker upwelling signature noticed near the northern tip of Somalia (10°N) also likely linked to the initial phase of the formation of GW. This early spin-up of GW coincide with the arrival of annual Rossby waves radiated from the west coast of India and therefore, considered to be one of the driving mechanisms for the formation of this gyre^18^. As the summer monsoon commences by June, the Findlater Jet peaks along the Somali coast leading to intensify upwelling all along the coast with D22 shoals around 50 m near the coast. By July, upwelling further intensifies along the coast and in the northern part surface temperature fall below 20 °C, a decrease of ~10 °C from that of the May. Further, in addition to the coastal upwelling, thermocline also shoals offshore at ~57°E for 8°N and 10°N section owing to the return flow of GW. This is the time when both the gyral systems, the SG and GW, also strengthen considerably and thereby help to intensify the upwelling further along the northern flank of the gyres. As a result, D22 penetrate the surface to form a strong cold wedge in the northern part off the Somalia. However, as the summer monsoon peaks during August, D22 starts to deepen considerably in all the sections. For example, at 8°N, the thermocline (D22) close to the coast deepened from 20 m in June/July to more than ~80 m during August, a deepening of about 60 m; indicating a possible downwelling over the major part of the Somali coast despite the strong upwelling favourable alongshore winds. Thermocline continues to deepen further in the month of September. Note, however, that while thermocline deepens over major part of the Somali coast, temperature of the upper water column cools progressively over the span of the Indian summer monsoon. In other words, the upwelling zones are restricted locally to the two gyral systems in the north and south and downwelling prevails over the central stretch of the Somali coast which itself accounts for 60% of the total length of the Somali coast.

### Windstress curl and Rossby waves

**Figure 7:**
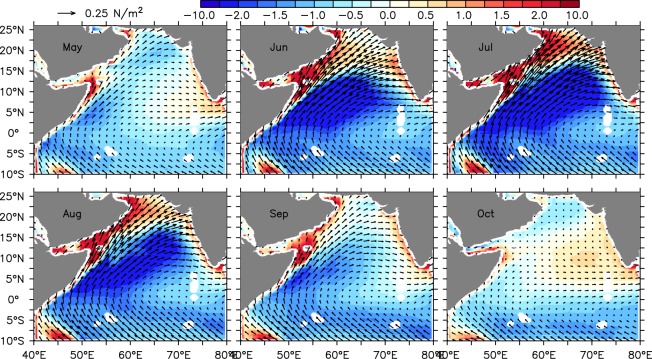
Climatological windstress curl (shaded) and windstress (vectors) derived from TropFlux data during 1993–2014. The vectors are plotted for every 6th grid.

Winds start to blow across the equator along the western boundary sometime in May (Fig. 7). This time winds are southwesterly in the northern Arabian Sea and predominantly alongshore off the southwestern part of the Indian coast. As a result, the windstress curl is negative over most part of the Arabian Sea, except over a narrow band along the coast of Somalia and Arabia and in the southeastern Arabian Sea. By June, southwesterly winds strengthen and blow across the Arabian Sea leading to a much stronger negative curl over the interior part of the Arabian Sea south of 15°N. During this period, the positive windstress curl is confined to the northern part of the Arabian Sea and to a narrow band hugging the coast of Somalia, Arabia and the west coast of India. The negative curl over the interior Arabian Sea further intensifies during July and persists till late September. By October, as the summer monsoon winds start to withdraw from the Indian continents, the windstress curl switches its sign from negative to positive over the interior basin.

**Figure 8:**
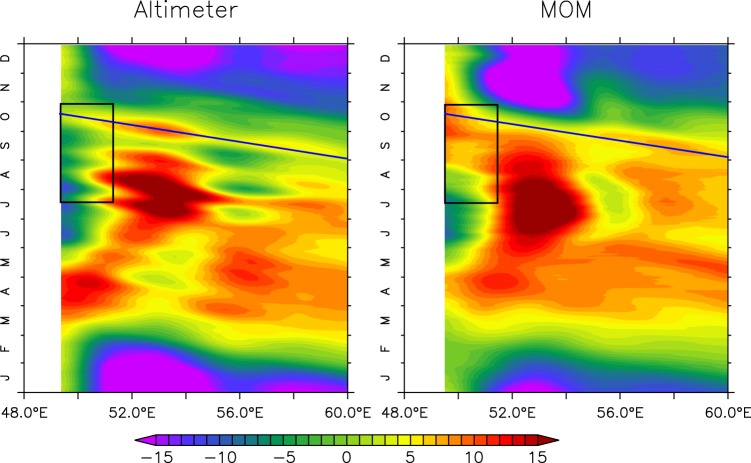
Hovmoller diagram of sea level anomaly from altimeter (left) and model (right) along 7°N exhibits westward phase propagation of downwelling Rossby waves. The black straight lines represent the phase lines and the black boxes marked the region where remotely forced Rossby waves intercept the coastal signal to elevate the sea level there. Note here that sea level anomaly from altimeter is calculated based on fixed datum, whereas model calculates sea surface height as the deviation from *z* = 0 level. In order bring both the datasets in same reference level, basin mean (averaged over Arabian Sea) are removed from the corresponding data sets.

Nevertheless, during summer monsoon the alongshore winds are poleward off the Somalia coast and therefore, are upwelling favorable. On the other hand, the strong negative windstress curl over large parts of the interior Arabian Sea results in strong Ekman pumping (negative vertical velocity) over the interior of the basin (Fig. S2). This leading to an elevated sea level (Figs S3 and S4) in the interior Arabian Sea. This signature can also be seen in the form of deeper thermocline as the sea level and thermocline are inversely correlated^27^. Subsequently, these downwelling signals then propagate westward with an approximate speed of 25 cm/s and interfere with the coastal signals along the coast of Somalia (Fig. 8). A significant aspect of the Fig. 8 is that the slope of the phase lines are consistent in the observation and model. Moreover, they are consistent with those of westward propagating Rossby waves. The dispersion relation of Rossby wave is

1 $$\sigma =\frac{\beta {k}_{n}}{{k}_{n}^{2}+{l}_{n}^{2}+{f}^{2}/{c}_{n}^{2}},$$

and therefore the group speed can be found as

2 $$\frac{\partial \sigma }{\partial {k}_{n}}=\beta \frac{\mathrm{(2}{k}_{n}^{2}-{r}_{\circ }^{-2})}{{\mathrm{(2}{k}_{n}^{2}+{r}_{\circ }^{-2})}^{2}},$$

here *k*_*n*_(=*l*_*n*_) and *c*_*n*_ are the wave number and the characteristic (Kelvin wave) speed of the *n*'th baroclinic mode. ${r}_{\circ }^{2}={c}_{n}^{2}/{f}^{2}$ represents the local Rossby radius of deformation and *f* represents the Coriolis parameter. Table 1 shows the values of Rossby wave speed with period *P* = 2*π*/*σ* and wavelength *λ*_*n*_ = 2*π*/*k*_*n*_ for the first three baroclinic modes. Since the fetch of the negative windstress curl is about the width of the Arabian Sea (i.e. ~30°) and the period is annual and therefore, excite those baroclinic modes which fit better with this prescribed length and time scale. As can be seen from the Table 1, none of the first three baroclinic mode will excite efficiently. Nevertheless, second and third mode will excite better compared to the first mode owing to the comparable length scale with the windstress curl. This is also evident as the observed Rossby wave speed can only be explained by a combination of all three modes.

**Table 1:** The theoretical group speed of the Rossby waves calculated based on the equation 2.

P	Mode 1 (*c*_*n*_ = 264 cm/s)	Mode 2 (*cn* = 166 cm/s)	Mode 3 (*cn* = 105 cm/s)
365 days	GS = 50 cm/s; *λ*_1_ = 144°	GS = 19 cm/s; *λ*_2_ = 56.5°	GS = 7 cm/s; *λ*3 = 22.5°

Once the westward propagating Rossby wave fronts intercepts with the coast of Somalia the zonal velocity associated with these waves get canceled at the boundary. The only possible way to cancel the negative zonal velocity at the boundary is by generating equatorward *β*-plane downwelling Kelvin wave along the coast which is against the prevailing upwelling by the alongshore winds. Earlier Shankar *et al*.^28^ showed a similar interior ocean response that drives a downwelling equatorward current of magnitude close to 100 cm/s along the coast of Somalia (see Figure 24 of their paper) using a linear model. This feature is more conspicuous during late July and August when these westward signals get further intensified by the Rossby waves generated from GW. Note here that, while the coastal waters tend to upwell, driven by the strong alongshore local winds via offshore Ekman transport, the remotely forced downwelling signal tends to suppress the upwelling along the coast. Therefore, the thermocline structure off the Somali coast is determined by the relative strength of the local winds and remotely forced Rossby waves: in regions where strength of the currents are much stronger, e.g. northern flanks of SG and GW, water upwells and along C_SEC_, where the surface currents are weaker, downwelling signal dominates. Also, as the negative windstress curl strengthens during June-August, the thermocline along C_SEC_ deepens to more than 100 m. Note, however, that Rossby waves generated at the interior ocean takes about 1–2 months to reach the coast and thereby, deepening of the thermocline is seen only by late July or early August i.e. lagged by about a month from the peak in the negative windstress curl.

### Cause of surface cooling

**Figure 9:**
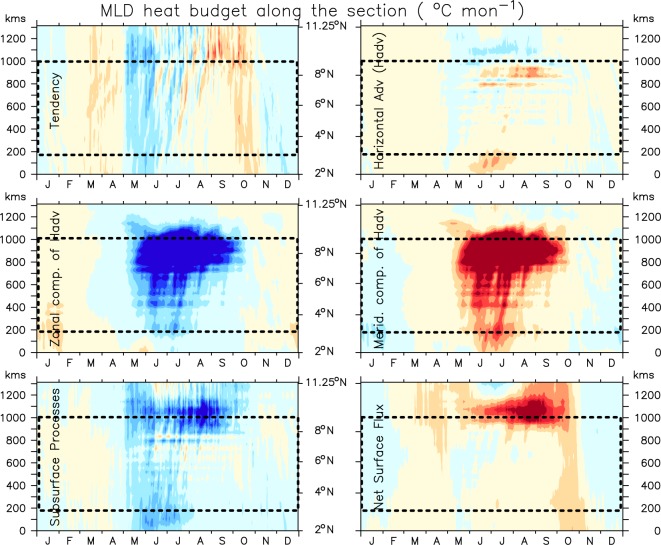
Evolution of each term of the climatological heat budget of the mixed layer given in Eq. 3 along the alongshore section. The negative temperature tendency during summer explained by the subsurface processes and net surface heat flux. Note that the heat budget is first computed over the interannual time series and then the climatology is computed over 1993–2014 period. Here dashed box represents central part of the section.

**Figure 10:**
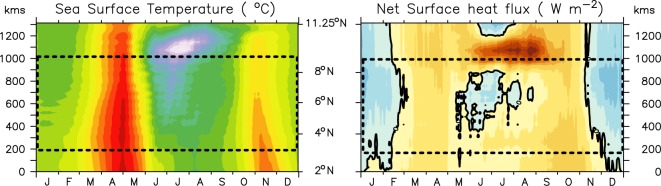
Climatological model simulated SST (left) and model derived net surface fluxe along the alongshore section. The black contour represents zero values. A similar plot for the net surface heat flux from JOFURO and TropFlux are shown in Fig. S5. Here dashed box represents central part of the section.

In the previous section, we discussed the mechanisms that force downwelling along the coast of Somalia despite having strong upwelling favourable alongshore winds. However, it still remains unclear that how surface water show strong cooling not only at the upwelling pockets, but all along the coast of Somalia. In order to understand that we have carried out a mixed layer heat budget of the model solution for the alongshore section off the Somali coast. The mixed layer heat budget is governed by the following equation:

3 $$\frac{\partial T}{\partial t}=-{{\rm{\Delta }}}_{H}T+\frac{{Q}^{\ast }}{\rho {C}_{p}}+R,$$

here, *T* is the averaged potential temperature of the mixed layer, Δ_*H*_*T* is the horizontal advection integrated over the mixed layer, Q^*^ is the net surface thermal flux corrected for shortwave penetration below the mixed layer, *ρC*_*p*_ is specific heat capacity of the sea water and *R* represents the contribution of all other processes including vertical advection and entrainment into the mixed layer (hereafter will refer as subsurface processes). Figure 9 shows the contribution of each heat budget components–to the observed upper ocean cooling along the alongshore section of 1000 m isobath. As the Inter-tropical convergence zone (ITCZ) lays over the equatorial belt during March-April, the surface temperature along the Somali coast steadily increases to ~30 °C off Somalia coast. The overlying winds are much weaker during this period which help in maintaining a positive SST tendency. By early May, as the winds begin to blow parallel to the coast, a sharp SST cooling is observed along the entire stretch of Somalia coast (Fig. 10). This observed low SST at the beginning of June persists throughout the summer monsoon with occasional warming/cooling as a response to the local monsoon intraseasonal variations. The SST tendency term remain predominantly negative during the entire summer monsoon months in the N_SEC_ and S_SEC_ with occasional switch to positive values in the C_SEC_. The occasional interchange of sign in temperature tendency may likely be linked to intrasesonal variability of the monsoon winds. Our analysis suggests that the horizontal advection helps to warm the surface layer along the major part of the Somali coast except in the north where it helps to cool the SST during the entire monsoon. A split up of this term into zonal and meridional advection exhibits a strong contrast among them. While the meridional advection, driven by the strong alongshore currents, brings enormous amount of heat into this region, particularly in the C_SEC_, the major portion of this heat gets advected away by the zonal advection. This results in a net weak warming tendency in the C_SEC_. In other words, the amount of water transported away by the offshore currents, gets replaced with warm water by the northward current along the coast and therefore, does not contribute much to the surface cooling here. In the N_SEC_, however, meridional velocity is close to zero and the zonal velocity field transport the coastal waters away thus helps in cooling the SST there. As a result, N_SEC_ shows strong cooling by the subsurface processes dominated by upwelling associated with GW as was seen in shoaling of the thermocline in Figs 5 and 6. A similar subsurface processes driven cooling owing to the SG can also be seen in S_SEC_. Interestingly, in the C_SEC_, especially towards the northern part of it, subsurface processes shows a significant cooling despite the fact that thermocline of the C_SEC_ deepens over the course of summer monsoon. Had the subsurface process driven cooling was dominated by upwelling, as is widely perceived, the thermocline of this region would have shoaled. Thus, the nature of subsurface processes that drive the observed cold surface temperature in the C_SEC_ are not yet clear and therefore, will be revisited again in the next section. Finally, the contribution of net surface heat flux is mostly positive along the entire section except during winter, during May in the south of GW and during May-July in the C_SEC_. This negative tendency in the C_SEC_ is primarily contributed by the negative (or near zero) net heat surface flux (Figs 10 and S6), which is comparable with the observed net heat flux based on TropFlux and JOFURO data sets (Figs S5 and S6).

Further analysis suggests that latent heat flux is strongly negative (~250 W/m^2^) over the C_SEC_ driven by large evaporation due to strong southwesterly winds and thus, determines the near zero net heat flux there (Fig. S7). Moreover, the strong positive net surface flux over the cold wedge of the GW (Figs 10 and S6) is found to be determined by the positive sensible heat flux (more than 100 W/m^2^) into the ocean (Fig. S7) driven by the strong temperature gradient between warm air temperature and very cold sea surface temperature.

## Upwelling vs. Entrainment

The idea here is to compare the relative strength of vertical entrainment mixing and upwelling (Ekman pumping) that dominates the subsurface processes. Instantaneous wind stirring, scaled as the friction velocity, causes vertical entrainment mixing through vertical shear instability of wind driven horizontal currents and surface buoyancy. This deepens the mixed layer and mixes the warm surface water with cold thermocline waters, resulting in the net cooling of the mixed layer. Whereas Ekman pumping generates vertical upwelling, and the subsurface water shoals into the mixed layer and cools the surface waters. In both these processes, the observable aspect is the same, i.e. the surface waters cools. But the mixed layer depth deepens during entrainment whereas it shoals during upwelling and hence, are driven by different dynamics. In the coming section, we explore which of these subsurface processes support the observed cooling off Somalia coast, particularly in the C_SEC_.

**Figure 11:**
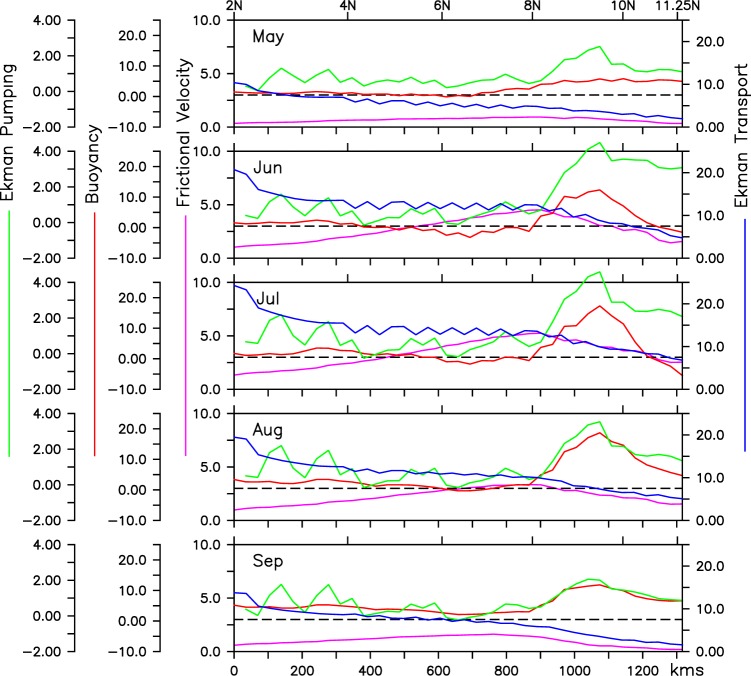
Climatological Ekman transport (blue; 10^3^ kg m^−1^ s^−1^), Ekman pumping (green; m day^−1^), cube of Frictional velocity (magenta; 10^−6^ m^3^ s^−3^) and buoyancy flux (red; kg m^−2^ s^−1^) along the alongshore section. Here, Ekman transport and Ekman pumping velocities are calculated based on climatological TropFlux winds. The black dotted line represents zero crossing for buoyancy flux and Ekman pumping. The shaded region in left and right represent S_SEC_ and N_SEC_.

We have analysed the surface flux of the turbulent kinetic energy (TKE) that drives the entrainment, which is a function of the sum of the cube of the frictional velocity and the surface buoyancy flux. The frictional velocity is defined as u_*_ = (*τ*/*ρ*_*o*_)^(1/2)^, where *τ* is surface wind magnitude and *ρ* is the density of the mixed layer. The surface buoyancy flux (*B*) is defined as a sum of the flux associated with the surface heat and fresh water flux and is given by *B* = (*α*/*C*_*p*_)*F*_*net*_ + *βρS*(*E* − *P*). Here, *α* and *β* are the thermal expansion and haline contraction coefficient, respectively. *C*_*p*_ is the heat capacity of the sea water, *F*_*net*_ is the net surface heat flux, *S* is the sea surface salinity and (*E* − *P*) represents evaporation minus precipitation. Additionally, we also compared Ekman pumping (*w*_*ek*_) which is a function of windstress curl (*w*_*ek*_ = *curl*(*τ*)/*f*) and crosshore Ekman transport (*EK*_*tr*_) which is dependent on alongshore windstress (*EK*_*tr*_ = *τ*_*ashore*_/*f*) during the summer months along the coast of Somalia (Fig. 11).

As stated above, Ekman mass transport is positive and transporting water offshore with an average rate of 10^4^ kg m^−1^s^−1^ with maximum in the south and decreases almost linearly in the north in accordance with the strength of the alongshore wind components. In S_SEC_, *EK*_*tr*_ crosses more than 2 × 10^4^ kg m^−1^s^−1^ during the peak monsoon season. Additionally, here, *w*_*ek*_ also show positive values during early summer and the thermocline shoals (Fig. 5). This indicates that the cooling observed in the S_SEC_, as shown in the previous section, is dominated by wind driven upwelling, which acts as a combination of strong offshore Ekman transport and upwelling favorable Ekman suction. Notably, weaker frictional velocity and a stable stratification (represented by positive Buoyancy flux) precludes the possibility of entrainment in the southern part of the section.

On the other hand, C_SEC_ exhibits a very different dynamics. While offshore Ekman transport is considerably strong in this part, the Ekman suction (positive *w*_*ek*_) is weak or close to zero. This is in agreement with the observed deepening of the thermocline in this region dominated by the downwelling favorable Rossby waves. Further, during June–July, owing to the intense net surface heat loss, buoyancy flux also turns negative and thus, leads to cooler and heavier surface water which then sink to enhance mixing at the bottom of the mixed layer. Additionally, as the frictional velocity also peaks up in this part of the section, in the presence of negative surface buoyancy, enhanced mixing occur in the surface layers to entrain subsurface water into the mixed layer. This leads to the deepening of the surface mixed layer and thermocline (D22), which is further enhanced by the the arrival of Rossby waves from the interior Arabian Sea. To sum up, the surface mixed layer water entrain into the cold subsurface layers and mixes with it and thus helps to cool the surface water. But unlike in a typical upwelling scenario, here due to entrainment, the thermocline deepens.

In contrary, along the N_SEC_, the buoyancy flux show a strong stratification with the minimum frictional velocity and therefore, does not support the possibility of entrainment in this region. Furthermore, as stated earlier, Ekman transport is also weakest in this part of the section. Interestingly, Ekman pumping velocity is strongest in this region and crosses more than 4 m/day during the peak monsoon, indicating that windstress curl induced Ekman pumping may contribute significantly in the formation of GW front, and the associated cooling in this region. Nevertheless, influence of nonlinear eddy driven divergence for the generation of GW and its associated upwelling can not be neglected.

## Discussion

The above analyses, using observations and model simulations, show a prominent downwelling feature in over ~60% of Somali coast line (the central section, C_SEC_) during peak summer monsoon. We find that the upwelling is confined only to early phase of the summer monsoon and later within the small pockets of northern flanks of the two gyres, SG and GW. Interestingly, in spite of the downwelling over major part of the Somali coast, SST shows a nearly uniform cooling all along the Somali coast.

Our analysis suggests a far reaching consequence at the way we look at the possible future changes in the Somali upwelling region. Bakun^19^ indicates that owing to the enhanced land-sea thermal gradient, in the warming climate, winds along the major EBUS will intensify and thus will increase the upwelling intensity. Afterwards, many researchers looked at the possible future changes in the upwelling zones, but reported contradictory conclusions primarily owing to the discrepancies in the used wind product and varying period of study^29,30^. A similar contradictory conclusions exist for the Somali region as well: while some suggest an increase in the summer upwelling^31,32^, few others suggested otherwise^33,34^. Here we show that there are multiple factors that drive the thermocline structure and resultant increase/decrease in SST along the Somali coast. While the alongshore winds are strongly upwelling favorable all along the coast, the remotely generated downwelling Rossby waves forced by the offshore negative windstress curl try to deepen the thermocline in the central region of the Somali coast. As a result, a major portion of the Somali coast downwell and the strong upwelling is only seen along the northern flank of the strong offshore currents associated with SG and GW. As a result, even though the surface cooling is uniform along the coast, the forcing mechanisms for such homogeneous cooling varies from south to north along the coast. While in the upwelling regions SST is primarily determined by the cold, upwelled subsurface water; in the downwelling region, it is entrainment aided by net negative buoyancy flux and strong wind energy input that contribute to lower SST. In short, future projection of Somali upwelling region only based on alongshore wind driven Ekman transport might be erroneous as opposed to the EBUSs and therefore, demands detailed analysis.

Further, during summer, cold surface temperature off the coast of Somalia and Arabia create much needed thermal gradient for moisture transport for the Indian summer monsoon. It is reported that during 1980–2014 the strength of the Findlater Jet enhanced^35^ and future projections based on CMIP models suggest that it may further intensify in the warming climate^36^. Notably, at the same time, negative windstress curl over the interior Arabian Sea suggest a positive trend^37^, favouring upper ocean warming by deepening the thermocline. Since, strengthening of summer monsoon winds helps in enhancing the thermal gradient across the Arabian Sea and therefore, in combination with increasing upper ocean heat content, favouring an enhanced moisture transport to the Indian land masses. Thus, providing a positive feedback to the Indian Summer monsoon over India. On the other hand, downwelling signals forced by the interior negative windstress curl ultimately deepen the thermocline along the Somali coast and therefore, impact the upwelling along the Somali coast to negatively feedback to the land-sea thermal gradient over this region. Further, this reduced upwelling can warm the western Arabian Sea which may lead to spatial westward shift in the monsoon depression and thereby will effect the rainfall over India^38^. These contrasting oceanic processes are not yet well studied in the context of the Indian summer monsoon rainfall, but, are important for the better prediction of future change in the rainfall over south Asia.

Furthermore, Somali upwelling region is considered to be the upwelling branch of the shallow overturning circulation (also known as cross-equatorial cell) of the Indian Ocean that play a vital role in the redistribution of heat in the upper water column of the Indian Ocean across the equator^39^. As shown here, upwelling along this coast is very limited to the early part of the monsoon and in small eddy driven regions, thus may not be much important to close the over turning circulations across the equator as was believed earlier. This opens up a question whether upwelling off Oman and Indian coast plays a bigger role in closing this cross equatorial circulation. Further study on this is needed to understand its dynamics and implication to the regional climate.

Another interesting aspect of our modeling analysis was the positive Latent heat flux (~40–60 w/m^2^) input into the ocean over the GW cold wedge during monsoon period, which is quite unusual in normal oceanic conditions. Hence, we verified this model derived latent heat flux (LHF) field with a satellite derived LHF product, JOFURO and another synthesis product, TropFlux (Figure not shown), and found similar positive LHF into the ocean across all the datasets. The theoretical reason for such an unusual feature can be explained based on bulk aerodynamic formula used for LHF estimation. As per the bulk formulae, LHF is proportional to the product of surface wind speed and the humidity gradient between sea surface and at 10 m height and goes in the direction of humidity gradient. Once the summer monsoon commences, wind speed strengthens, and the only possibility to make a downward LHF flux is by making an inverse near surface humidity gradient (i.e. near surface air more moist than sea surface air). The near surface winds off Somalia are traveling from relatively warmer southern hemisphere/Equator and hence are warmer and therefore, carry large amount of moisture. Once they cross over the cold wedge of the GW, the surface air becomes cooler, but due to the effect of subsurface processes SST cools much more and hence creates an inversion in the near surface humidity structure. As the surface air itself cools down, it can no longer hold the carried moisture anymore and hence condenses most probably in the form of fog. This is a tricky situation to explain as this feature happened due to the inability to accommodate such an unusual situation in the bulk formulae or it is in fact a physical reality is yet to be verified. Direct measurement of fluxes (covariance fluxes) are needed to ascertain this process further.

Finally, Somali region is considered to be one of the most productive place of the Indian Ocean driven by huge amount nutrient upwelled from subsurface water^10^. However, we show a very complex thermocline structure along the coast of Somali as the upwelling is limited to the northern part and in some extent in the southern part of the Somali coast and the large middle part exhibits entrainment driven by downwelling. How this varied physical subsurface process influence the upper water column concentration of nutrients is, therefore, require further study. In a recent study based on satellite derived SST Kankan *et al*.^40^ showed that Somali coast exhibits one of weakest SST front among the other persistent frontal systems of the north Indian Ocean. They also added that this weaker front off Somalia exists only during early part of the summer monsoon and not during the entire season. As the frontal system is often associated with productive regions, weaker frontogenesis off Somalia contradicts the strong productivity reported earlier. In the absence of direct observations, numerical modelling is the only possible approach in this scenario. We will put more light onto the bio-physical processes in a separate study.

In summary, we have explored the mechanisms that drive the cold surface temperature along the Somali coast during summer monsoon period and showed that upwelling is not the sole cause of this, in fact entrainment and net surface flux controls the SST over major part of this region. In other words, this paper not only put more light on the oldest known upwelling system of Indian Ocean, but probably, raise many more questions related to the possible future changes of regional climate and productivity.

## Methods

### Model

We use a regional Indian Ocean model based on Modular Ocean Model (MOM4p1^41^). This model is used extensively earlier by many^42–44^. The model spans over 30–120°E and 30°S–30°N with uniform 0.25° horizontal resolution and 40 vertical levels with 5 m resolution in top 60 m^42^. The model equations are discretized using generalized orthogonal coordinate on a staggered Arakawa B-grid with hydrostatic, volume conserving Boussinesq approximation. The vertical mixing is based on K-profile parametrization scheme (KPP^45^) and for the horizontal mixing a Smagorinsky type combination of bihermonic and Laplacaian mixing is used. The model topography is based on modified ETOPO2^46^ with minimum depth is set to 15 m. Salinity and Temperature is relaxed to climatological values with a 30-day time scale in the 5° sponge layer along the open southern and eastern boundaries. No restoration is applied anywhere else in the model domain.

#### Forcing

Model is forced by surface fluxes obtained from various sources. The radiation (shortwave and longwave), momentum (windstres) fluxes and 2 m specific humidity and air temperature are obtained from TropFlux data^47,48^. Whereas, the surface air pressure and rainfall is prescribed from NCEP^49^. To determine shortwave penetration into the ocean water column climatological chlorophyll data from SeaWifs (http://nomads.gfdl.noaa.gov) is used. Freshwater river input^50,51^ is introduced into the top 10 m of the water column.

#### Model run

Ocean model is initially spun-up for 10 years using the climatological forcing derived for the period 1993–2014 using the forcing mentioned above. Then the model run forward using interannal forcing for 1993–2014. The result presented here are based on climatological fields derived based on the model interannual simulations for 1993–2014.

### Data

The monthly mean values of sea surface temperature is obtained from the tropical rainfall measuring misson’s (TRMM) microwave imager (TMI) (http://apdrc.soest.hawaii.edu/data/data.php) and the TRMM rainfall data is downloaded from http://daac.gsfc.nasa.gov/precipitation. The third generation data sets from Japanese Ocean Flux Data Sets with Use of Remote Sensing Observations (J-OFURO) is obtained from https://j-ofuro.scc.u-tokai.ac.jp/en/ and the TropFlux data are available from https://www.incois.gov.in/tropflux/. The reanalysis data from Estimating the Circulation and Climate of the Ocean (ECCO) product is retrieved from https://ecco.jpl.nasa.gov/, the data from Operational Ocean Reanalysis System version 4 (ORAS4) are obtained from https://climatedataguide.ucar.edu/climate-data/oras4-ecmwf-ocean-reanalysis-and-derived-ocean-heat-content and the Simple Ocean Data Assimilation (SODA) version 2.2.4 data are retrieved from http://apdrc.soest.hawaii.edu:80/dods/public_data/SODA/soda_pop2.2.4. Gridded climatological temperature and salinity data from World Ocean Atlas 2013 (WOA13) are downloaded from https://www.nodc.noaa.gov/OC5/woa13/ and the North Indian Ocean Atlas (NIOA) are avaiable in http://www.nio.org/index/option/com_nomenu/task/show/tid/2/sid/18/id/229.

### Analysis and plots

The data analysis and all the plots presented here are made using Ferret, PyFerret and Matlab.

## Supplementary information

Supplementary Figures
