# Author Correction: Annihilation of the Somali upwelling system during summer monsoon

**DOI:** 10.1038/s41598-019-54632-x

**Published:** 2019-12-06

**Authors:** Abhisek Chatterjee, B. Praveen Kumar, Satya Prakash, Prerna Singh

**Affiliations:** ESSO- Indian National Centre for Ocean Information Services, Hyderabad, India

Correction to: *Scientific Reports* 10.1038/s41598-019-44099-1, published online 20 May 2019

In Figures 9 and 10, the colour scale is missing. The correct Figures 9 and 10 appear below as Figures 1 and 2 respectively.

**Figure 1:**
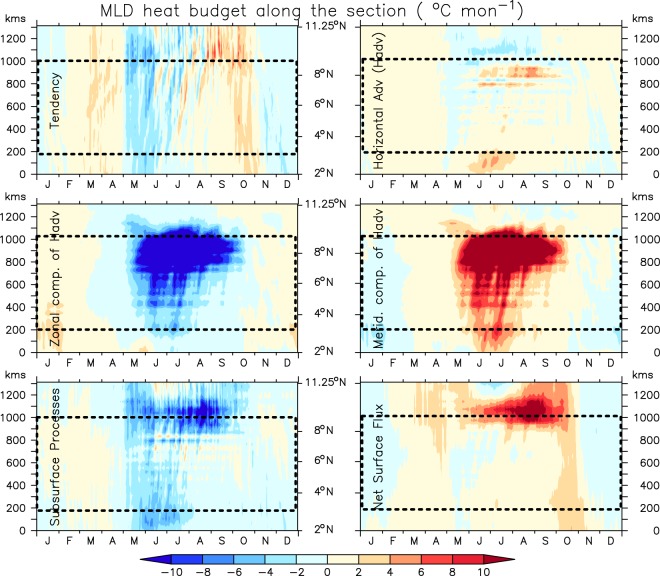
.

**Figure 2:**
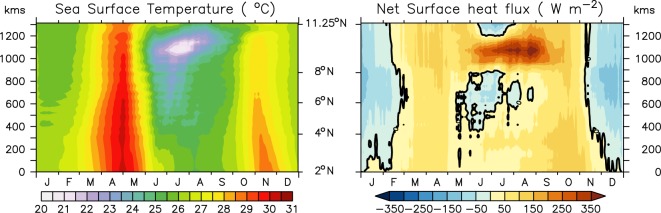
.

