# Supplementary material for: Annihilation of the Somali upwelling system during summer monsoon
Source: Sci Rep. 2019 May 20;9:7598. doi: 10.1038/s41598-019-44099-1 (PMC6527695; doi:10.1038/s41598-019-44099-1)
Supplement: Supplementary file 1 — Supplementary Figures [file 41598_2019_44099_MOESM1_ESM.pdf]

Annihilation of the Somali upwelling system during summer monsoon

Abhisek Chatterjee[1,*], Praveen Kumar B[1], Satya Prakash[1], and Prerna Singh[1]

[1]ESSO- Indian National Centre for Ocean Information Services, Hyderabad, India
[*]abhisek.c@incois.gov.in

ABSTRACT

This document includes supplementary figures.

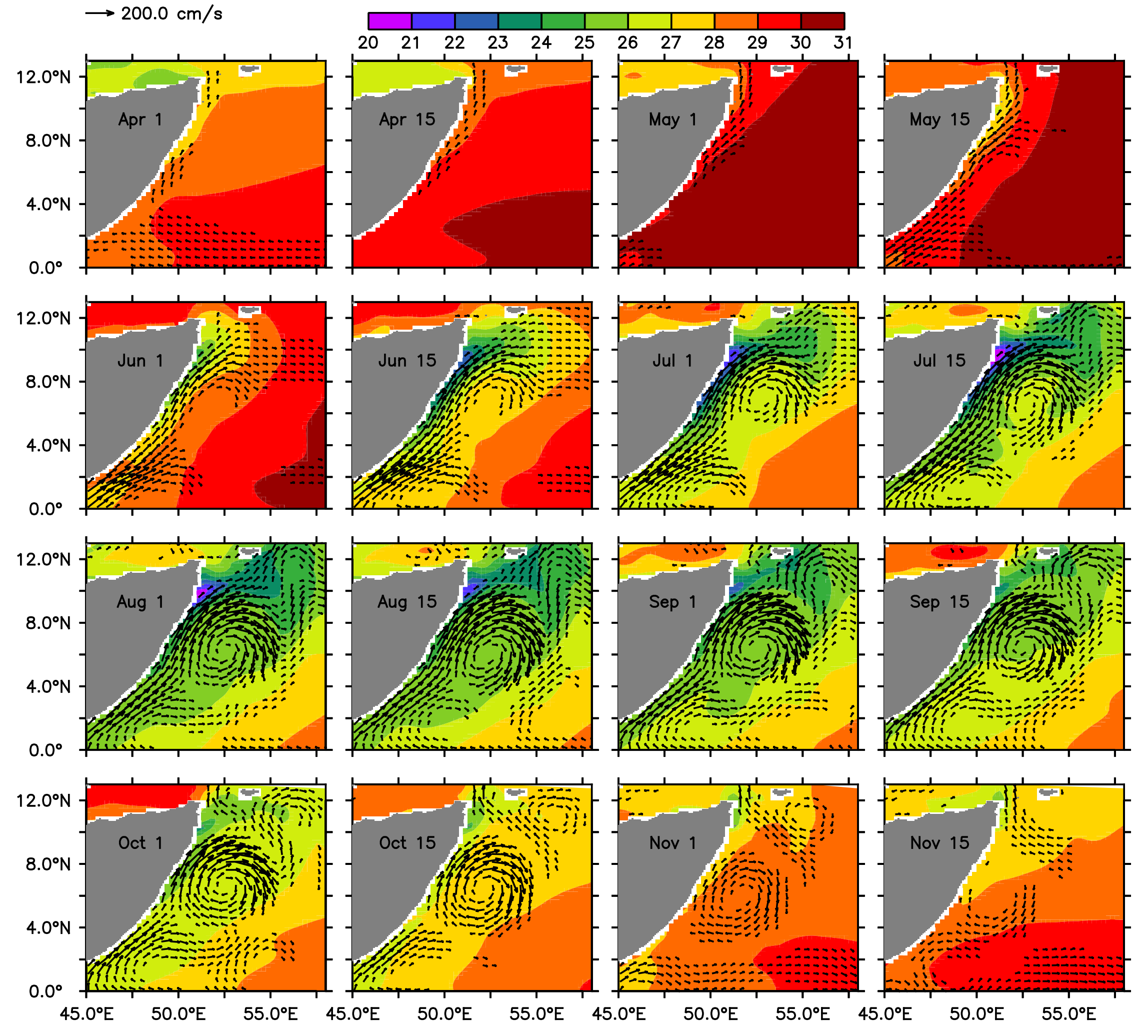

Figure S1. Fortnightly map of model simulated climatological SST (°C) and surface currents. Note that current vectors of less than 50 cm/s are masked out to show the stronger currents only and the vectors are plotted for alternate grids.

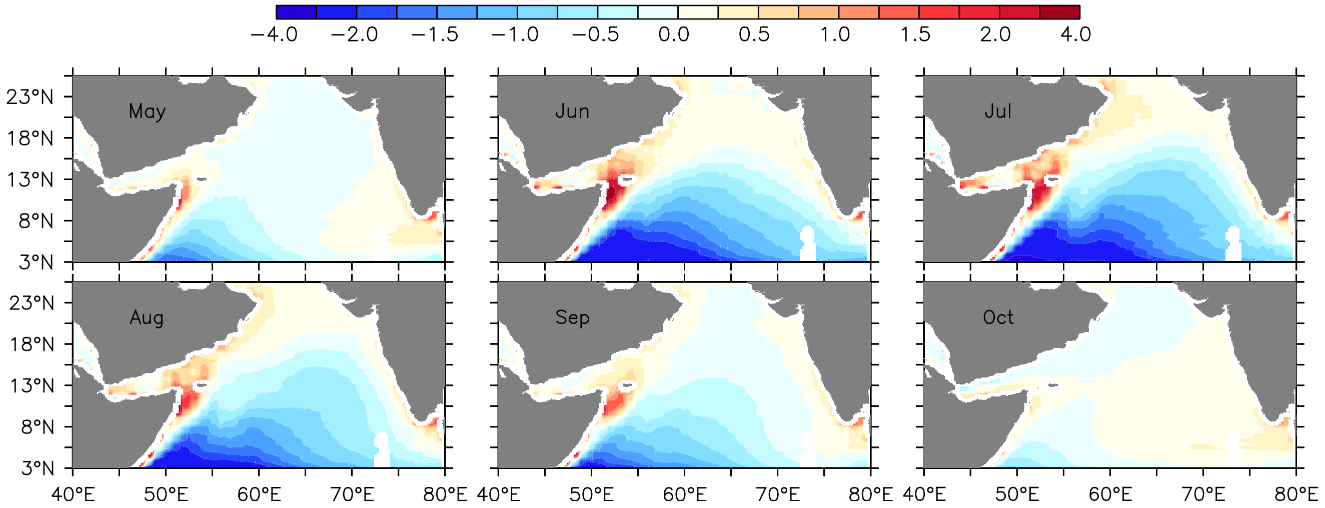

Figure S2. Monthly mean Ekman pumping velocity (m day^{-1}) calculated based on climatological TropFlux windstress. Strong downward Ekman pumping velocities are evident off Somalia driven by downwelling favorable wind stress curl. This offshore downwelling signals radiate downwelling Rossbywaves towards the coast which ultimately deepen the thermocline along the Somali coast. However, strong positive Ekaman pumping in the northern tip of Somalia helps to upwell water there.

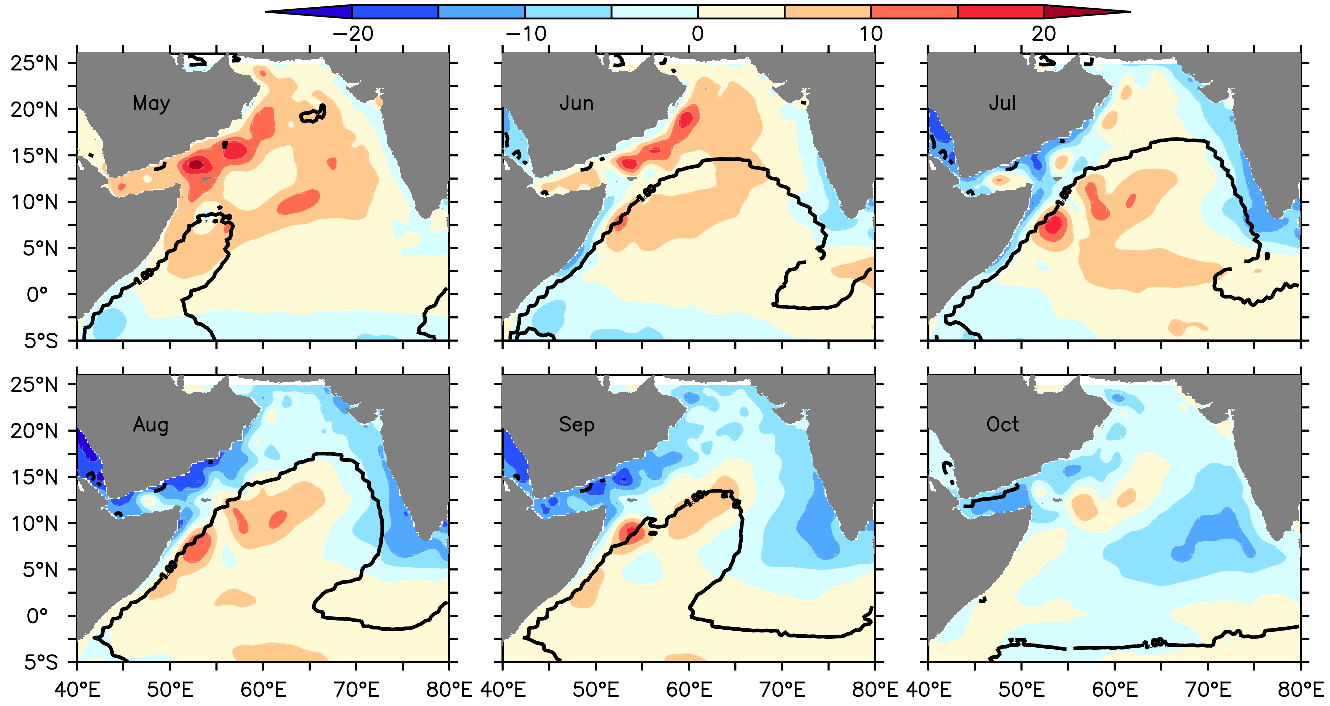

Figure S3. Shaded colour represents monthly mean climatological sea level anomaly from altimeter and the black contour showing the region with negative $(-1 \times 10^7 \mathrm{Nm}^{-3})$ windstress curl from Tropflux windstress. Note here that the basin averaged sea level is removed from the altimeter anomaly.

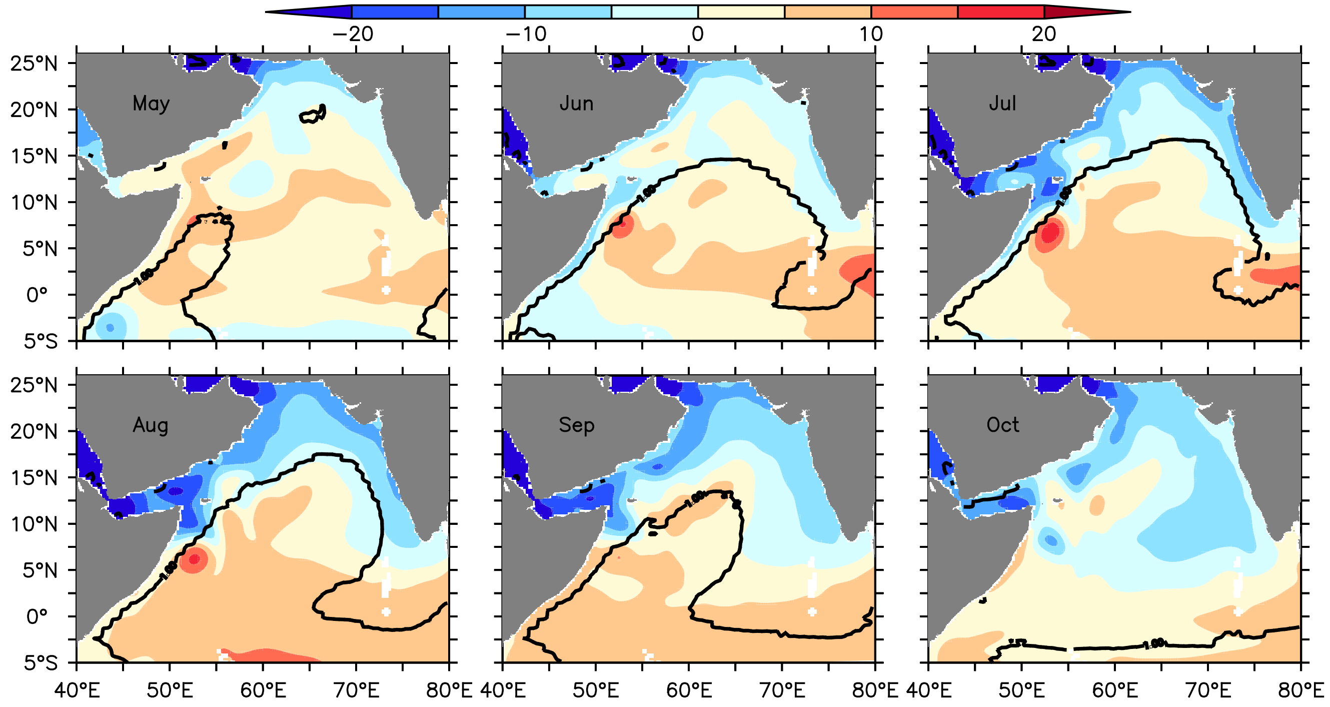

Figure S4. Same as Figure S3 except the sea level anomaly is from model simulated climatology. Note here that the basin averaged sea level is removed from the model simulated anomaly.

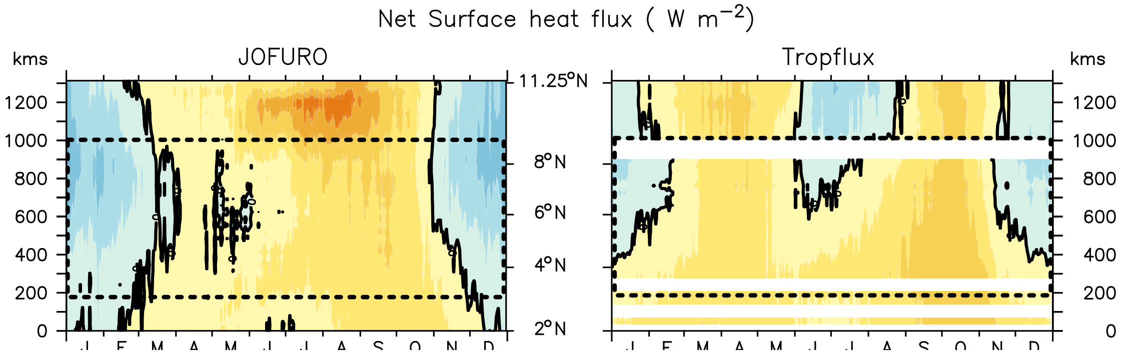

Figure S5. The net surface heat flux from JOFURO (left) and tropflux (right) along the section at 1000 m isobath off Somali coast. Note here that absence of strong positive net flux over the northern part of the section in Tropflux is owing to its coarse grid resolution and also the white stripes in the north and south are due to land mask of the data. Here dashed box represents central part of the section.

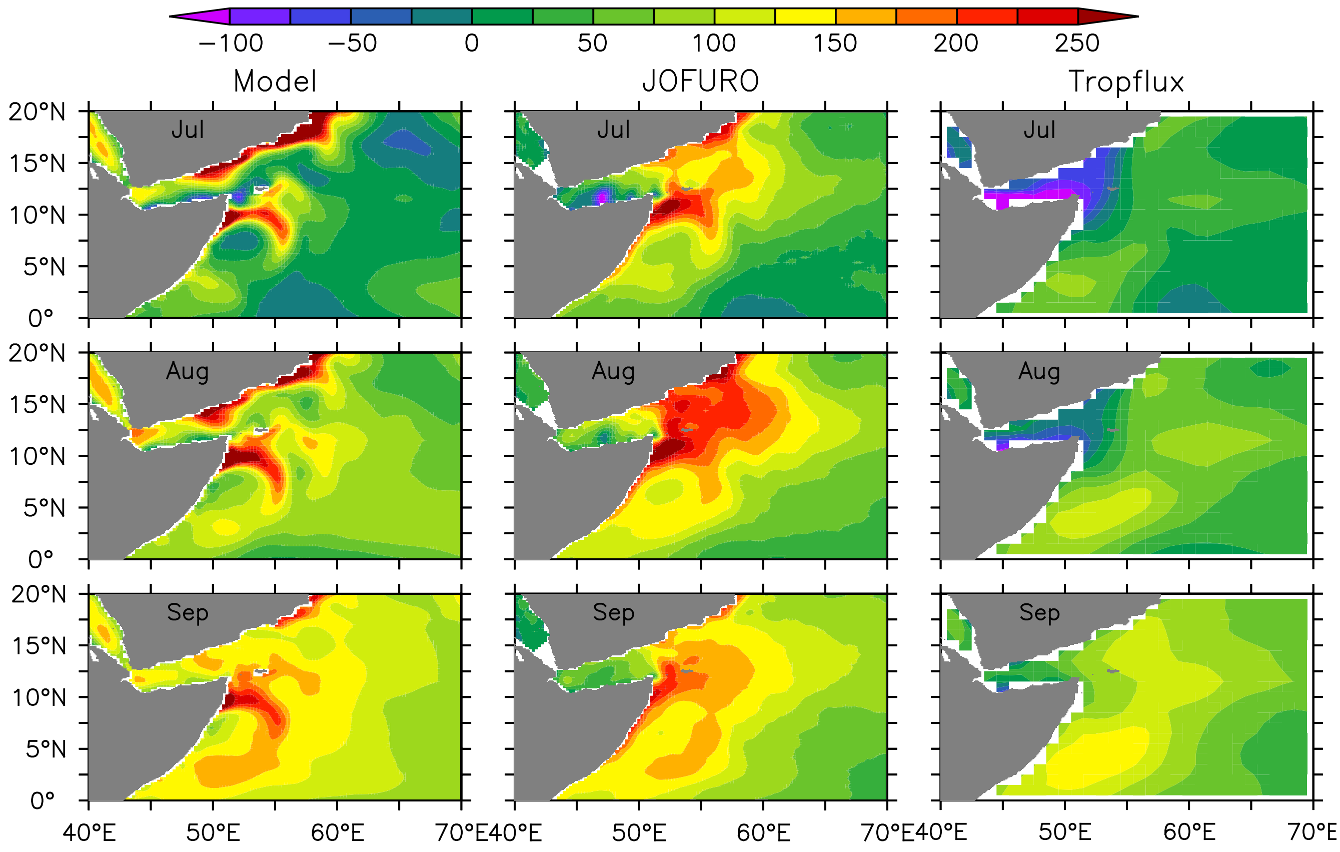

Figure S6. Monthly averaged climatological net surface heat flux from Model (left), JOFURO (middle) and TropFlux (right) for the western Arabian Sea. Note that despite the model is forced with TropFlux data model is able is capture the spatial structure of net surface heat flux as evident in the observation (JOFURO). The prognostic SST evolution in the model allows a better net surface heat flux than is actually provided by TropFlux. This is possible in the model as it is forced with only longwave, shortwave and specific humidity along with winds and model calculates the net surface flux dynamically based on prognostic SST simulation. Note also that absence of strong positive net flux over the northern part of the Somali region (along the GW front) in TropFlux is owing to its coarse grid resolution.

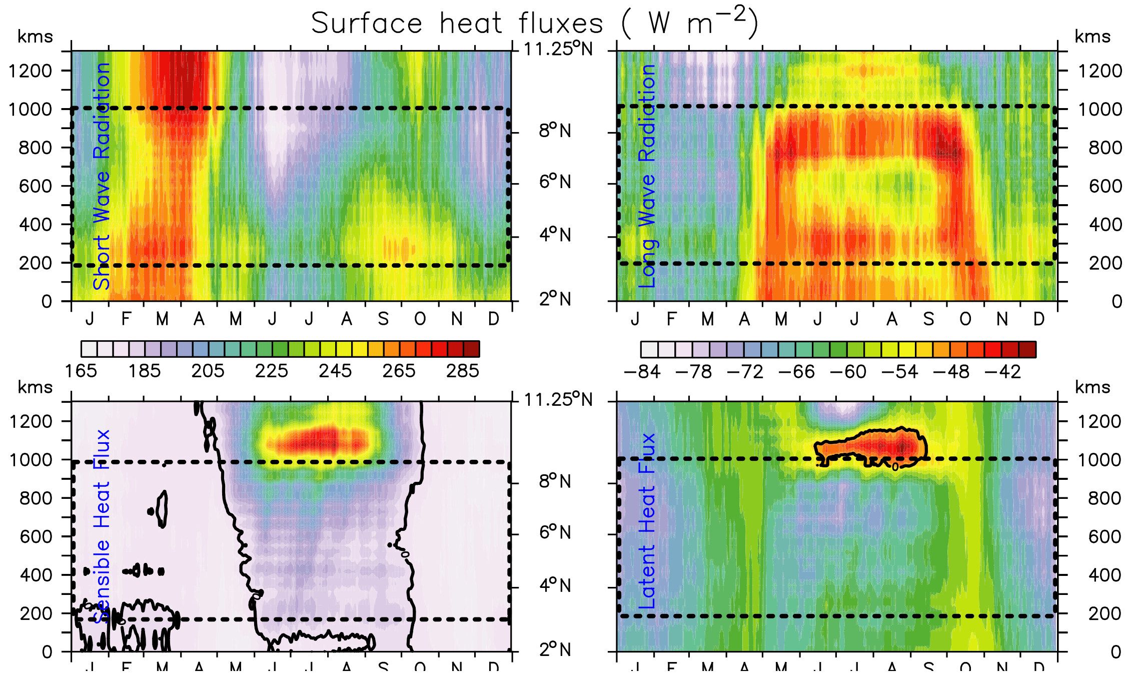

Figure S7. Model simulated climatological heat flux components along the section at 1000 m isobath off Somali coast. Here dashed box represents central part of the section.
